# Pharmacological targeting of mitochondrial function and reactive oxygen species production prevents colon 26 cancer-induced cardiorespiratory muscle weakness

**DOI:** 10.18632/oncotarget.27748

**Published:** 2020-09-22

**Authors:** Ashley J. Smuder, Brandon M. Roberts, Michael P. Wiggs, Oh Sung Kwon, Jeung-Ki Yoo, Demetra D. Christou, David D. Fuller, Hazel H. Szeto, Andrew R. Judge

**Affiliations:** ^1^Department of Applied Physiology and Kinesiology, University of Florida, Gainesville, FL, USA; ^2^Department of Physical Therapy, University of Florida, Gainesville, FL, USA; ^3^Department of Kinesiology, University of Connecticut, Storrs, CT, USA; ^4^Social Profit Network Research Lab, Alexandria LaunchLabs, New York, NY, USA

**Keywords:** diaphragm, cachexia, heart, SS-31, elamipretide

## Abstract

Cancer cachexia is a syndrome characterized by profound cardiac and diaphragm muscle wasting, which increase the risk of morbidity in cancer patients due to failure of the cardiorespiratory system. In this regard, muscle relies greatly on mitochondria to meet energy requirements for contraction and mitochondrial dysfunction can result in muscle weakness and fatigue. In addition, mitochondria are a major source of reactive oxygen species (ROS) production, which can stimulate increased rates of muscle protein degradation. Therefore, it has been suggested that mitochondrial dysfunction may be an underlying factor that contributes to the pathology of cancer cachexia. To determine if pharmacologically targeting mitochondrial dysfunction via treatment with the mitochondria-targeting peptide SS-31 would prevent cardiorespiratory muscle dysfunction, colon 26 (C26) adenocarcinoma tumor-bearing mice were administered either saline or SS-31 daily (3 mg/kg/day) following inoculation. C26 mice treated with saline demonstrated greater ROS production and mitochondrial uncoupling compared to C26 mice receiving SS-31 in both the heart and diaphragm muscle. In addition, saline-treated C26 mice exhibited a decline in left ventricular function which was significantly rescued in C26 mice treated with SS-31. In the diaphragm, muscle fiber cross-sectional area of C26 mice treated with saline was significantly reduced and force production was impaired compared to C26, SS-31-treated animals. Finally, ventilatory deficits were also attenuated in C26 mice treated with SS-31, compared to saline treatment. These data demonstrate that C26 tumors promote severe cardiac and respiratory myopathy, and that prevention of mitochondrial dysfunction is sufficient to preclude cancer cachexia-induced cardiorespiratory dysfunction.

## INTRODUCTION

Cachexia is a debilitating consequence of cancer, most prevalent in patients with gastrointestinal, pancreatic, lung and colorectal cancers [1]. This condition adversely affects patient outcomes as a result of the continuous loss of fat and muscle mass, leading to compromised physical function, independence and quality of life [2]. Indeed, body weight loss in individuals with cancer cachexia is accompanied by cardiac muscle wasting and significant inspiratory (i.e., diaphragm) muscle weakness, which often present initially as fatigue, shortness of breath and exercise intolerance, but can compromise survival by advancing into ventilatory and/or heart failure [3–5]. Currently, there are no effective therapeutic countermeasures to combat cancer cachexia, thus elucidating new drug targets is critical.

Although the underlying mechanisms driving cancer-induced cardiorespiratory muscle weakness remain unknown, emerging evidence reveals that cancer promotes mitochondrial damage, resulting in alterations to electron transport chain complex activity, impaired ATP synthesis and the production of reactive oxygen species (ROS) [6]. Additionally, mitochondrial impairment in muscle tissue has been shown to cause profound functional and structural disorganization [7–9]. In fact, impaired mitochondrial function was demonstrated to precede the development of cachexia in Lewis lung carcinoma tumor-bearing mice, which supports the premise that mitochondrial dysfunction may be critical to the development of cancer cachexia [10]. Importantly, the early development of mitochondrial dysfunction promotes mitochondrial ROS production and the oxidation of muscle contractile proteins which upregulates the activation of proteolytic pathways and accelerates muscle protein breakdown, leading to disruption of muscle contractile properties and muscle wasting [11–13]. Therefore, targeting early changes in mitochondrial function and ROS production could be instrumental in preserving cardiac and skeletal muscle mass and function.

In this regard, SS-31 is a mitochondria-targeted peptide that concentrates within the inner mitochondrial matrix where it binds to cardiolipin and improves mitochondrial function through facilitation of electron transfer, inhibition of cytochrome c peroxidase activity and reduction of proton leak [14]. Preclinical investigation into the efficacy of SS-31 administration for the preservation of mitochondrial function during conditions that promote cardiac and diaphragm muscle weakness (e.g., chemotherapy myotoxicity, mechanical ventilation, heart failure, sepsis, etc.) has revealed a significant interaction between mitochondrial dysfunction and muscle weakness [15–19]. Therefore, we tested the hypothesis that maintenance of mitochondrial quality via SS-31 administration would be sufficient to prevent cardiorespiratory muscle dysfunction in C26 tumor-bearing mice.

## RESULTS

### Biological response to SS-31 treatment and C26 tumors

Body weight did not differ among groups prior to the initiation of the experimental protocol (Table 1). All mice were euthanized ~26–28 days following C26 cell inoculation. This time point corresponded with largest tumor size reaching 1.5 cm in diameter. Final body weight and final tumor-free body weight were significantly reduced in the TB-SALINE mice compared to all other groups, reflective of cachexia. In TB-SS-31 mice, final body weight was maintained compared to non-tumor-bearing mice. However, when tumor weight was subtracted TB-SS-31 mice weighed significantly less than the CON-SALINE mice. Note that tumor weight was identical for the TB-SALINE and TB-SS-31 groups. The decline in body weight in the TB-SALINE mice was accompanied by a reduction in heart, gastrocnemius, plantaris and extensor digitorum longus (EDL) weight compared to non-tumor-bearing mice. Muscle weight in the TB-SS-31 group was preserved for all muscles except the final heart weight, which was significantly reduced compared to the CON-SALINE mice.

**Table 1:** Biological response to treatments

	CON-SALINE	CON-SS-31	TB-SALINE	TB-SS-31
**Initial Weight (g)**	22.75 ± 0.27	22.48 ± 0.24	22.70 ± 0.22	22.98 ± 0.18
**Final Weight (g)**	26.01 ± 0.51	24.65 ± 0.62	21.78 ± 1.01^*^	24.72 ± 0.55
**Final Weight (tumor-free) (g)**	26.01 ± 0.51	24.65 ± 0.62	19.45 ± 0.94^*^	22.40 ± 0.44^^^
**Tumor Mass (g)**	—	—	2.32 ± 0.16	2.32 ± 0.30
**Heart Weight (mg)**	121.8 ± 3.6	119.3 ± 3.6	94.1 ± 3.9^#^	106.3 ± 3.5^^^
**Tibialis Anterior (mg)**	48.2 ± 1.6	47.6 ± 1.5	36.7 ± 1.0^#^	38.4 ± 1.0^#^
**Gastrocnemius (mg)**	139.4 ± 5.2	138.3 ± 4.0	108.0 ± 10.8^#^	114.3 ± 6.2
**Plantaris (mg)**	17.4 ± 1.3	16.4 ± 0.8	12.0 ± 0.9^#^	13.9 ± 1.3
**EDL (mg)**	8.8 ± 0.3	8.7 ± 0.7	7.5 ± 0.4	7.6 ± 0.6

Differences in body weight, tumor weight and muscle weight between experimental groups. Values are presented as means ± SEM. ^*^significantly different versus all groups (*p* < 0.05); ^ significantly different versus CON-SALINE (*p* < 0.05); ^#^significantly different versus CON-SALINE and CON-SS-31 (*p* < 0.05).

### Preservation of cardiac function in C26 cachexia with SS-31 treatment

**Figure 1:**
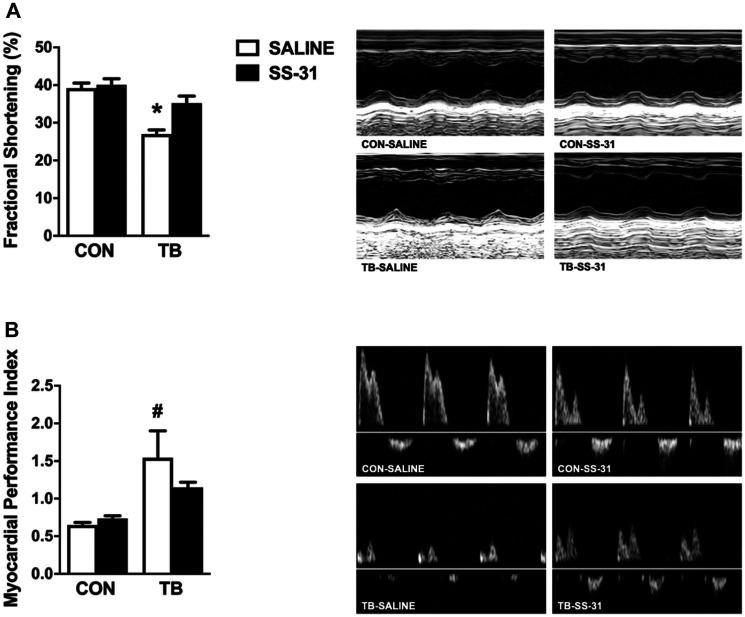
Cardiac function. Heart left ventricular function for control (CON) and tumor-bearing (TB) mice treated with saline (SALINE) or SS-31 assessed by: (**A**) fractional shortening percentage and (**B**) myocardial performance index. Values are presented as means ± SEM. Representative echocardiographic images are shown to the right of the graphs. ^*^significantly different versus all groups (*p* < 0.05); ^#^significantly different versus CON-SALINE and CON-SS-31 (*p* < 0.05).

Cardiac function was assessed for all experimental groups via echocardiography. Assessment of left ventricle (LV) systolic function demonstrated a significant reduction in fractional shortening in the TB-SALINE mice compared to all other groups (Figure 1A). The reduction in fractional shortening in the TB-SALINE mice was a result of increased LV internal systolic diameter (LVIDs) (Table 2). In addition, evaluation of LV global systolic and diastolic function revealed a significant increase in myocardial performance index (MPI) in TB-SALINE mice, but not TB-SS-31 mice, compared to the CON-SALINE and CON-SS-31 groups (Figure 1B). The increased MPI was due to elongation of the isovolumetric contraction and relaxation time and an impaired ejection phase. Finally, analysis of LV wall thickness also revealed significant thinning of the septal wall during systole in TB-SALINE mice compared to non-tumor-bearing mice, and of the posterior wall during systole in TB-SALINE mice compared to all groups (Table 2).

**Table 2:** Cardiac left ventricle wall thickness and inner diameter

	CON-SALINE	CON-SS-31	TB-SALINE	TB-SS-31
**SWTd (mm)**	0.81 ± 0.07	0.78 ± 0.04	0.74 ± 0.04	0.74 ± 0.04
**SWTs (mm)**	1.31 ± 0.07	1.33 ± 0.05	1.08 ± 0.06^#^	1.16 ± 0.05
**PWTd (mm)**	0.96 ± 0.07	0.83 ± 0.05	0.79 ± 0.04	0.80 ± 0.05
**PWTs (mm)**	1.44 ± 0.04	1.30 ± 0.04	1.11 ± 0.04^*^	1.29 ± 0.04
**LVIDd (mm)**	3.58 ± 0.20	3.58 ± 0.15	3.44 ± 0.19	3.58 ± 0.09
**LVIDs (mm)**	2.13 ± 0.16	2.15 ± 0.12	2.68 ± 0.13^#^	2.32 ± 0.07

Differences in cardiac left ventricle septal wall thickness during diastole (SWTd) and systole (SWTs), posterior wall thickness during diastole (PWTd) and systole (PWTs), and left ventricular internal diameter during diastole (LVIDd) and systole (LVIDs) between experimental groups. Values are presented as means ± SEM. ^*^significantly different versus all groups (*p* < 0.05); ^#^significantly different versus CON-SALINE and CON-SS-31 (*p* < 0.05).

### Diaphragm and ventilatory function are maintained in C26 cachexia with SS-31 treatment

**Figure 2:**
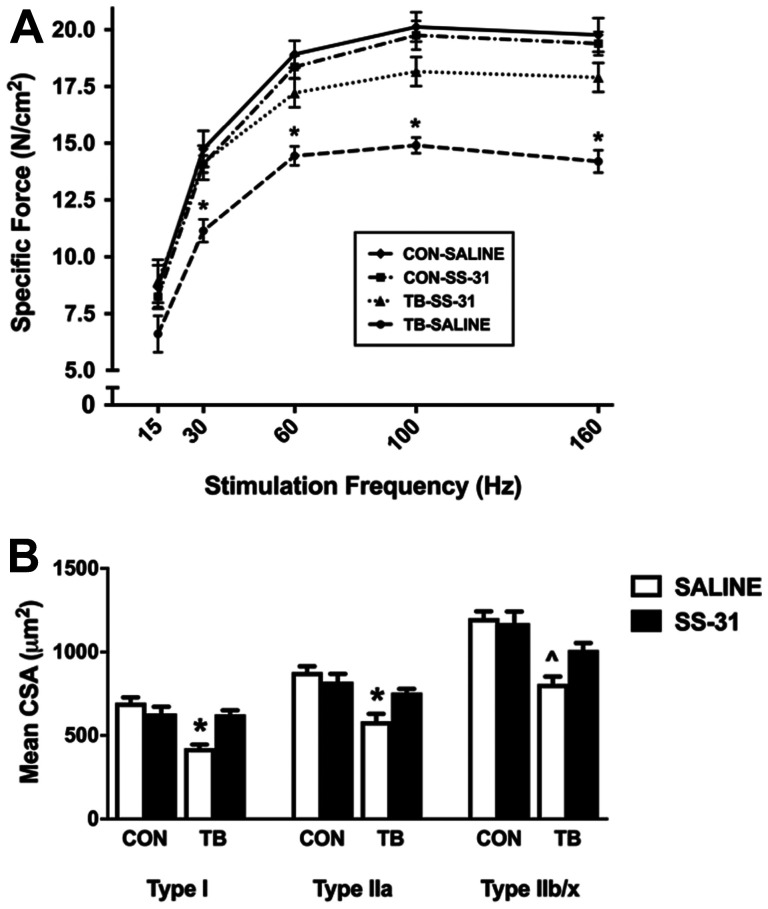
Diaphragm muscle function and fiber size. Diaphragm muscle (**A**) force-frequency response and (**B**) cross-sectional area for control (CON) and tumor-bearing (TB) mice treated with saline (SALINE) or SS-31. Values are presented as means ± SEM. ^*^significantly different versus all groups (*p* < 0.05); ^^^significantly different versus CON-SALINE (*p* < 0.05).

C26 cancer cachexia has been demonstrated to depress the *in vitro* diaphragm muscle force-frequency response and to reduce the maximal specific force production by 30% [20]. Our results clearly support these previous findings as diaphragm specific force production in TB-SALINE mice was significantly blunted at stimulation frequencies of 30–160 Hz compared to CON-SALINE mice (Figure 2A). Similarly, EDL contractile function was also significantly reduced in TB-SALINE mice compared to all other groups at stimulation frequencies of 60–200 Hz (Supplementary Figure 1A). Importantly, no functional impairment of the diaphragm or EDL muscle was observed in the TB-SS-31 mice.

In addition to contractile dysfunction, skeletal muscle fiber atrophy is a hallmark of cachexia [20, 21], and here we demonstrate a significant reduction in mean cross-sectional area (CSA) of Type I, Type IIa and Type IIb/x diaphragm muscle fibers in the TB-SALINE mice compared to CON-SALINE mice (Figure 2B). Importantly, daily administration of SS-31 prevented the C26-induced atrophy of Type I and Type IIa fibers. Mean CSA of the EDL was also significantly reduced in the TB-SALINE mice, but preserved in TB-SS-31 mice (Supplementary Figure 1B).

**Figure 3:**
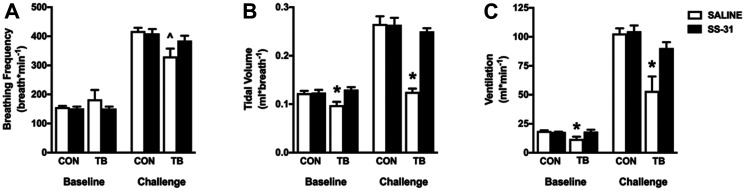
Respiratory function. Ventilatory pattern for control (CON) and tumor-bearing (TB) mice treated with saline (SALINE) or SS-31 during baseline (normoxic) and challenge (hypoxia) conditions. (**A**) breathing frequency; (**B**) tidal volume; and (**C**) minute ventilation. Values are represented as means ± SEM. ^*^significantly different versus all groups (*p* < 0.05); ^^^significantly different versus CON-SALINE (*p* < 0.05).

Diaphragm atrophy and dysfunction can compromise ventilation, and respiratory complications are a leading cause of mortality in cancer patients [22]. Using whole-body plethysmography to measure ventilation we show a significant reduction in tidal volume and minute ventilation, with no change in breathing frequency in the TB-SALINE mice compared to all other groups during normoxia (Figure 3A–3C). All mice responded to the 5-minute hypoxic challenge by increasing minute ventilation. However, the TB-SALINE mice were unable to increase inspiratory tidal volume during the challenge. The TB-SS-31 mice showed a robust increase in tidal volume, which was comparable to that of control mice.

### C26 tumors promote increased mitochondrial reactive oxygen species production and decreased mitochondrial oxidative capacity

**Figure 4:**
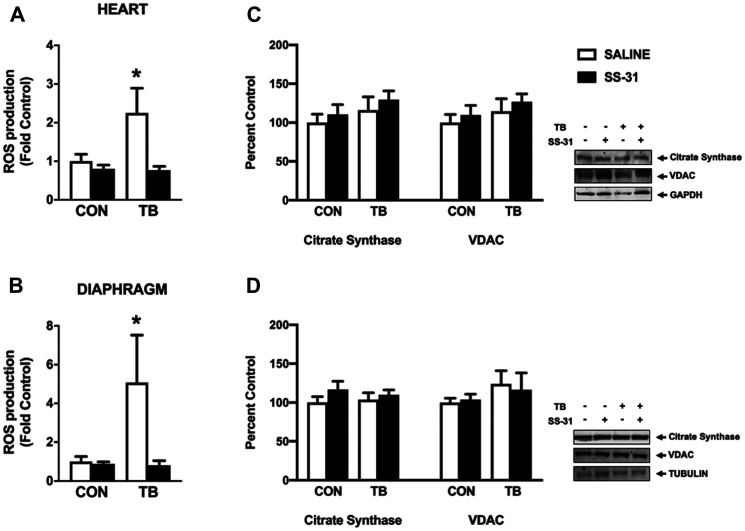
Mitochondrial reactive oxygen species (ROS) emission and protein expression. Mitochondrial ROS production from (**A**) heart and (**B**) diaphragm permeabilized muscle fiber bundles for control (CON) and tumor-bearing (TB) mice treated with saline (SALINE) or SS-31; and (**C**) heart and (**D**) diaphragm citrate synthase and VDAC protein expression for control (CON) and tumor-bearing (TB) mice treated with saline (SALINE) or SS-31. Values are represented as means ± SEM. Representative western blot images are shown to the right of the graphs. ^*^significantly different versus all groups (*p* < 0.05).

Permeabilized muscle fiber bundles from the heart and diaphragm were used to determine the effects of C26 tumors and SS-31 administration on mitochondrial ROS production and function. In this regard, increased ROS production coupled with a reduction in antioxidant capacity has been suggested as a contributing factor to the progression of cancer cachexia [8]. Our data establish that C26 tumors cause increased mitochondrial ROS emission in both the heart and diaphragm, and that the mitochondrial ROS emission in these tissues remains at basal levels in C26 tumor-bearing mice who receive SS-31 daily (Figure 4A and 4B). SS-31 administration to C26 tumor-bearing mice also resulted in maintenance of mitochondrial efficiency, as the respiratory control ratio (RCR) for both the heart and diaphragm was significantly depressed in the TB-SALINE animals compared to all other groups (Table 3). Moreover, the reduction in RCR occurred as a result of a decrease in state 3 respiration and an increase in state 4 respiration. Changes in mitochondrial volume were measured via western blot for citrate synthase and voltage-dependent anion channel (VDAC). No difference among groups existed for either protein in the heart or diaphragm (Figure 4C and 4D).

**Table 3:** Mitochondrial function

	CON-SALINE	CON-SS-31	TB-SALINE	TB-SS-31
**Heart**				
**State 3 (nmoles O_2_/mg/min)**	17.50 ± 1.12	16.76 ± 0.04	10.09 ± 0.54^*^	15.57 ± 0.85
**State 4 (nmoles O_2_/mg/min)**	2.52 ± 0.19	2.31 ± 0.13	3.45 ± 0.34^§^	2.78 ± 0.33
**RCR (State 3/State 4)**	7.09 ± 0.40	7.27 ± 0.36	3.09 ± 0.28^*^	5.87 ± 0.60
**Diaphragm**				
**State 3 (nmoles O_2_/mg/min)**	17.89 ± 1.02	17.21 ± 1.29	11.65 ± 0.95^#^	15.19 ± 2.05
**State 4 (nmoles O_2_/mg/min)**	2.46 ± 0.24	2.48 ± 0.16	3.75 ± 0.36^#^	2.79 ± 0.42
**RCR (State 3/State 4)**	7.58 ± 0.46	6.93 ± 0.21	3.17 ± 0.20^*^	5.82 ± 0.70

Cardiac and diaphragm muscle mitochondria state 3 respiration, state 4 respiration and respiratory control ratio (RCR). Values are presented as means ± SEM. ^*^significantly different versus all groups (*p* < 0.05); ^#^significantly different versus CON-SALINE and CON-SS-31 (*p* < 0.05). ^§^significantly different versus CON-SS-31 (*p* < 0.05).

### SS-31 treatment does not affect plasma TNFα, IL-1β or IL-6 levels in C26 tumor-bearing mice

**Figure 5:**
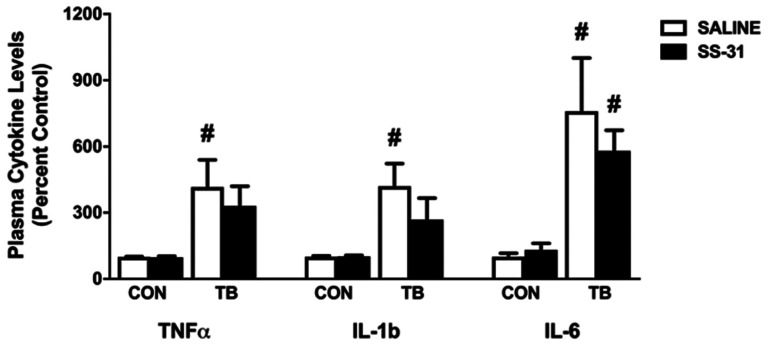
Circulating cytokines. Plasma cytokine levels of TNFα, IL-1b and IL-6 for control (CON) and tumor-bearing (TB) mice treated with saline (SALINE) or SS-31. Values are represented as means ± SEM. ^#^ significantly different versus CON-SALINE and CON-SS-31 (*p* < 0.05).

It is well-established that C26 cachexia alters the plasma levels of several soluble factors including TNFα, IL-1β and IL-6 which can accelerate the activity of key proteolytic pathways involved in the development of muscle weakness [23, 24]. In this study, we confirmed that plasma levels of TNFα, IL-1β and IL-6 are increased in TB-SALINE mice compared to CON-SALINE and CON-SS-31 (Figure 5). Of these cytokines, IL-6 remained significantly elevated in SS-31-treated C26 tumor-bearing mice compared to CON-SALINE and CON-SS-31 mice. TNFα and IL-1β levels in the TB-SS-31 animals were numerically elevated compared to CON-SALINE and CON-SS-31, but did not reach statistical significance.

### Differential expression of proteolysis markers in cardiac and diaphragm muscle of C26 tumor-bearing animals

**Figure 6:**
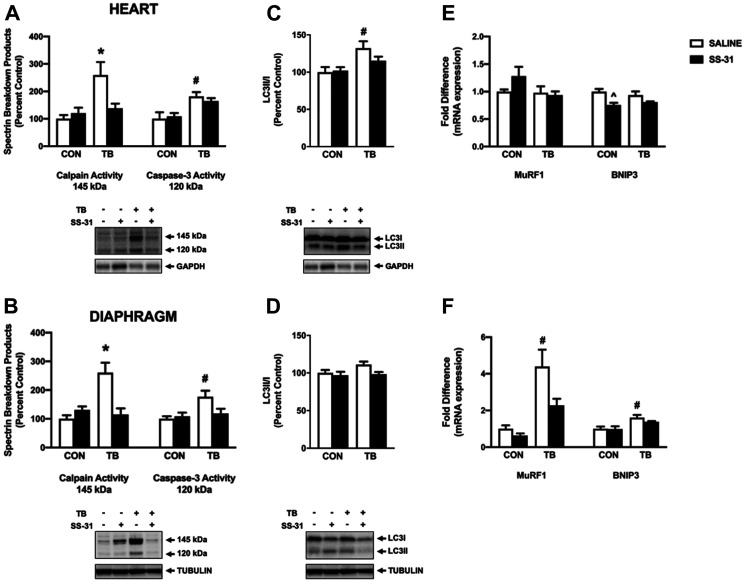
Markers of proteolytic activity. Western blot analysis of the calpain (145 kDa) and caspase-3 (120 kDa)-specific spectrin breakdown product in (**A**) heart and (**B**) diaphragm for control (CON) and tumor-bearing (TB) mice treated with saline (SALINE) or SS-31. Western blot analysis of LC3II/LC3I in (**C**) heart and (**D**) diaphragm for control (CON) and tumor-bearing (TB) mice treated with saline (SALINE) or SS-31. Gene expression of MuRF1 and BNIP3 in (**E**) heart and (**F**) diaphragm for control (CON) and tumor-bearing (TB) mice treated with saline (SALINE) or SS-31. Values are represented as means ± SEM. Representative western blot images are shown below the graphs. ^*^significantly different versus all groups (*p* < 0.05); ^#^significantly different versus CON-SALINE and CON-SS-31 (*p* < 0.05).

Enhanced proteolytic activity of both calpain and caspase-3 is required for cardiorespiratory muscle dysfunction in a variety of conditions [16, 25, 26]. Activity of calpain and caspase-3 was measured via western blot analysis of their specific αII-spectrin breakdown products (Figure 6A and 6B). Protein expression of the 145 kDa spectrin breakdown product revealed that calpain activity was elevated in the heart and diaphragm muscle of TB-SALINE mice compared to all groups. However, SS-31 administration to C26 mice abolished this increase in calpain activity in both tissues. Similarly, the 120 kDa product indicated a significant increase in caspase-3 activity in both the heart and skeletal muscle of TB-SALINE mice compared to both control groups. Caspase-3 activity did not differ between groups when compared to the TB-SS-31 mice.

Protein degradation by the autophagy/lysosomal system and the ubiquitin-proteasome system has been shown to increase in C26-tumor bearing mice [27, 28]. Our data show variation in the expression of markers of these proteolytic systems between heart and diaphragm muscle. Specifically, the ratio of LC3II to LC3I in the heart of TB-SALINE mice was significantly elevated compared to non-tumor-bearing animals (Figure 6C); whereas in the diaphragm there were no differences in the expression in this marker of autophagy (Figure 6D). Conversely, mRNA expression of the Bcl-2 protein family member BNIP3 and the E3 ubiquitin ligase MuRF1 were elevated in TB-SALINE mice compared to controls in the diaphragm (Figure 6E). In the heart, no differences existed among groups in the expression of MuRF1, and BNIP3 gene expression was reduced in the CON-SS-31 mice compared to CON-SALINE (Figure 6F).

## DISCUSSION

The findings from this study provide evidence that systemic administration of the mitochondria-targeted peptide SS-31 prevents cardiorespiratory muscle dysfunction in C26 tumor-bearing mice, independent of changes to tumor size or circulating levels of select cytokines. Our results showed that both saline and SS-31-treated tumor-bearing mice reached IACUC mandated tumor size endpoint 26–28 days post inoculation, but at that time tumor-free body weight was 15% greater in the SS-31-treated group compared to the saline-treated group. Muscle mass was also preserved in C26 mice treated with SS-31. While changes in food intake could contribute to our findings, pair-fed and cachectic C26 tumor-bearing mice showed significant differences in cardiorespiratory muscle function and muscle mass when compared [29, 30]. These data suggest that the effects to diaphragm and cardiac muscle were the result of tumor growth and SS-31 treatment.

Direct targeting of mitochondria with the SS-31 peptide is established to prevent cardiac and respiratory muscle weakness that occurs following a variety of conditions, including ventilator-induced diaphragm dysfunction, heart failure, sepsis and chemotherapy-related cachexia [15–19, 31]. Evidence from these studies indicates that perturbations to mitochondrial function can act as an upstream trigger in the activation of pathways which promote degradation of the muscle contractile apparatus resulting in muscle atrophy and weakness [15–19]. Here we add to these previous findings and demonstrate that daily treatment of C26 tumor-bearing mice with SS-31 protects against cardiorespiratory muscle dysfunction. Interestingly, the mechanisms for muscle protein breakdown between cardiac and diaphragm muscle appear to be differentially regulated in this study, as markers of autophagy/lysosomal system activation were upregulated exclusively within the heart. Conversely, the ubiquitin E3 ligase MuRF1 was elevated specifically within the diaphragm, and SS-31 treatment in C26 tumor-bearing mice did not alter the expression of these markers compared to non-tumor-bearing mice. Moreover, in agreement with previous reports, SS-31 administration to healthy control mice did not result in any adverse effects, and no differences existed between control groups for any dependent measure. Notably, cardiac contractile function was maintained in the SS-31-treated C26 mice independent of preservation of cardiac mass. While the mechanisms are unclear, this observation is consistent with findings by Tian et al. which revealed a significant reduction in absolute cardiac mass in C26 tumor-bearing mice and pair-fed mice compared to non-tumor-bearing mice; however, fractional shortening was maintained in pair-fed mice compared to C26 tumor-bearing mice [30].

Recently published work from Neyroud et al. demonstrated increased mitochondrial uncoupling and a reduction in mitochondrial respiratory capacity in the soleus muscle of cachectic C26 tumor-bearing mice [6]. In an extension of those findings, we show here compromised mitochondrial function in the heart and diaphragm of cachectic C26 tumor-bearing mice. Further, we show that SS-31 treatment preserved mitochondrial function in both cardiac and diaphragm muscle from C26 mice by preserving oxidative phosphorylation and preventing proton leak into the mitochondrial matrix. A similar positive influence of SS-31 on cardiac and skeletal muscle in heart failure was recently shown which implicated mitochondrial bioenergetics and redox signaling in the development of muscle dysfunction [31, 32]. This is important because increased proton leak can augment oxidant production within the mitochondrial matrix leading to a disruption of redox balance within the muscle tissue [33, 34]. Oxidative stress is a well-established upstream trigger sufficient to promote muscle catabolism and dysfunction in a variety of conditions [35]. Increased protein oxidation in muscle tissue results in alterations to structure and function, which enhances proteolytic breakdown by calpain, caspase-3, the ubiquitin-proteasome system and the autophagy/lysosomal system [36–38]. Oxidation of actin and tropomyosin has been demonstrated in heart failure and skeletal muscle weakness which likely contributes to contractile impairment [39–41]. In addition, increased ROS production can disrupt muscle contraction by altering calcium homeostasis. Muscle dysfunction associated with calcium channel functionality potentially occurs as a result of oxidation of the ryanodine receptor and subsequent calcium leak from the sarcoplasmic reticulum, as restoring calcium channel function in cancer patients prevented muscle weakness [42]. Further work is needed to determine the precise effects of SS-31 on myofibrillar proteins and calcium regulatory proteins, and their contribution to cancer-induced cardiorespiratory dysfunction.

In regards to cancer cachexia, oxidative stress has been identified as a critical mediator of muscle breakdown in pre-clinical models, which has led to clinical trials assessing the efficacy of antioxidant supplementation on patient lean body mass, fatigue and appetite [43]. Similar to an antioxidant, SS-31 is known to reduce oxidative damage to muscle tissue indirectly via preservation of mitochondrial function and also through its ability to directly scavenge mitochondrial ROS [44, 45]. Our results confirmed these effects as SS-31 prevented the cancer-induced supraphysiological production of ROS in cardiorespiratory muscles, which may also explain the reduction in several markers of proteolytic activity in SS-31 treated tumor-bearing mice. Currently, utilization of antioxidant therapy during cancer treatment remains controversial. Nevertheless, these findings warrant future preclinical studies to determine whether there are beneficial or adverse interactions of SS-31 supplementation in combination with cancer treatment.

Several pro-inflammatory cytokines, including IL-6 and TNFα, have been linked to the wasting phenotype in tumor-bearing hosts [23, 46–48]. Specifically, inflammatory cytokines released from the tumor, or from the host in response to the tumor, may engage their receptors on muscle cells and disrupt oxidative metabolism, proteostasis and/or muscle function [49]. Further, increased systemic inflammation has been postulated to promote the transmission of oxidative stress from diseased organs to muscle tissue [50]. However, a recent investigation into the factors driving cachexia-induced skeletal muscle function by VanderVeen et al. showed that increased muscle fatigue and decrements in mitochondrial biogenesis and content preceded development of cachexia in Apc^Min/+^ mice [51]. Additionally, no relationship was found between systemic IL-6 levels and muscle fatigue [51]. These findings were supported in the C26 mice treated with SS-31, as the preservation of both muscle and mitochondrial function was not associated with attenuated plasma IL-6 levels.

## MATERIALS AND METHODS

### Experimental animals

These experiments were performed in accordance with the guidelines for the Care and Use of Laboratory Animals. The Animal Care and Use Committee at the University of Florida approved these experiments. Eight-week old male CD2F1 mice (~20 g) were purchased from Charles River Laboratories (Wilmington, MA, USA) and were maintained on a 12:12 hour light: dark cycle and provided food and water *ad libitum*.

### Tumor inoculation and drug administration

C26 cells were obtained from the National Cancer Institute Tumor Repository (Fredrick, MD, USA). Cells were cultured in RPMI 1640 (Mediatech, Herndon, VA, USA) supplemented with 10% FBS, 100 U/ml penicillin and 100 μg/ml streptomycin at 37°C in a 5% CO_2_ humidified atmosphere. Mice were anesthetized with gaseous isoflurane and 5 × 10^5^ C26 cells, or 1 x PBS as a control, was subcutaneously (SC) injected into each flank. Cardiac function was assessed and muscles were harvested when the largest tumor diameter reached 1.5 cm (~26–28 days post-inoculation). SS-31 or saline (vehicle for SS-31) was administered daily (3 mg/kg; SC) beginning one day following inoculation.

### Echocardiography

Cardiac function was assessed via transthoracic echocardiography (Aplio XV, Toshiba Medical Systems, Tokyo, Japan) with a 12MHz transducer in animals anesthetized via inhaled isoflurane. Two-dimensional ultrasound images and M-mode tracings of the LV were obtained in the parasternal short-axis view at the level of the papillary muscles to determine LV wall thickness and systolic function. Fractional shortening was calculated using the following equation: (LV internal diastolic diameter (LVIDd) − LVIDs) × 100/LVIDd. MPI was assessed using pulse-wave Doppler at the level of the mitral valve leaflet tips and was calculated as: (isometric contraction time + isovolumetric relaxation time)/ejection time. Measurements were performed on five to ten cardiac cycles and averaged for each mouse.

### Histology

Sections from diaphragm and EDL muscles were cut at 10 μm using a cryotome (HM 550 Cryostat, Thermo Fisher Scientific, Waltham, MA, USA). For the assessment of muscle fiber type and CSA, muscle sections were incubated with primary antibodies for myosin heavy chain type I (A4.840, Developmental Studies Hybridoma Bank, Iowa City, IA, USA), myosin heavy chain type IIa (SC-71, Developmental Studies Hybridoma Bank) and dystrophin (RB9024, Thermo Fisher Scientific) followed by incubation with appropriate fluorescently conjugated secondary antibodies (Thermo Fisher Scientific). Images were captured using a monochrome camera (Qimaging Retiga) attached to an inverted fluorescent microscope (Zeiss Axiovert 200, Oberkochen, Germany). CSA was determined using Scion software (NIH, Bethesda, MD, USA).

### *In vitro* muscle contractile properties

A muscle strip, including the tendinous attachment at the central tendon and rib cage was dissected from the mid-costal region. The strip was suspended vertically between two lightweight plexiglass clamps with one end connected to an isometric force transducer (Dual-mode lever system, Aurora Scientific, Aurora, ON, Canada) within a jacketed tissue bath containing a Krebs-Hensleit solution equilibrated with 95% O_2_–5% CO_2_ gas. Additionally, the EDL muscle was also dissected and prepared for measurement of contractile properties. The force output was recorded via a computerized data acquisition system (LabView 8.6, National Instruments, Austin, TX). After a 15-min equilibration period, *in vitro* diaphragm contractile measurements were made. The muscle strip was stimulated along its entire length with platinum wire electrodes (S48 stimulator, Grass Instruments) to determine the optimum contractile length (L_o_). L_o_ was determined by systematically adjusting the length of the muscle and evoking single twitches. To measure the force-frequency response each strip was stimulated supramaximally with 120-V pulses at 15, 30, 60, 100 and 160Hz while at L_o_. The duration of each train was 500ms to achieve a force plateau. Contractions were separated by a two-minute recovery period. Diaphragm force production was normalized to specific optimal force. The total muscle CSA at right angles to the long axis was calculated by the following algorithm: total muscle CSA (mm^2^) = [muscle mass/(fiber length × 1.056)], where 1.056 is the density of muscle (in g/cm^2^). Fiber length was expressed in centimeters measured at L_o_.

### Whole-body plethysmography

A barometric whole body plethysmograph (Buxco, Wilmington, NC) was used to measure breathing in unanesthetized mice prior to sacrifice. Ventilatory data was collected and analyzed as previously described [20]. Briefly, a minimum of 30-minutes was allowed for acclimatization to the chamber, with a stable 5-minute period being used for baseline measurements (21% O_2_, 79% N_2_). Following baseline, mice were given a short respiratory challenge by exposure to a 5-minute period of hypoxia (10% O_2_, balance N_2_).

### Permeabilized muscle fibers

Small portions (~4 mg) of diaphragm muscle were dissected and placed in a plastic petri dish containing ice cold buffer X containing: 60 mM K-MES, 35 mM KCl, 7.23 mM K_2_EGTA, 2.77 mM CaK_2_EGTA, 20 mM imidazole, 0.5 mM DTT, 20 mM taurine, 5.7 mM ATP, 15mM phosphocreatine and 6.56 mM MgCl_2_, pH 7.1. The muscle fibers were gently separated to maximize surface area, and were permeabilized in buffer X with 50 μg/ml saponin by rotation for 30 min at 4°C. Permeabilized muscle fiber bundles were then washed in buffer Z containing: 110 mM K-MES, 35 mM KCl, 1 mM EGTA, 5 mM K_2_HPO_2_, 3 mM MgCl_2_, 0.005 mM glutamate, 0.02 mM malate and 0.5 mg/ml BSA, pH 7.1.

### Mitochondrial respiration

Mitochondrial respiration was measured via polarography in permeabilized muscle fiber bundles as previously described [16]. Briefly, water-jacketed respiration chambers were maintained at 37°C (Hansatech Instruments, King’s Lynn, UK). Each chamber contained 1 ml of respiration buffer containing: 110 mM K-MES, 35 mM KCl, 1 mM EGTA, 5 mM K_2_HPO_2_, 3 mM MgCl_2_·6H_2_O, 5 mg/ml BSA, and 20 mM creatine to saturate creatine kinase. Maximal ADP-stimulated respiration (state 3) was obtained using complex I substrates (i.e., 2 mM pyruvate and 2 mM malate) in the presence of 0.25 mM ADP and state 4 respiration was determined by adding 10 μg/mL oligomycin to inhibit ATP synthesis. The RCR was then calculated by dividing state 3 by state 4 respiration.

### Mitochondrial ROS emission

Hydrogen peroxide emission rate was measured from permeabilized heart and diaphragm muscle fiber bundles in a multi-well plate reader (SpectraMax, Molecular Devices, Sunnyvale, CA, USA) at 37°C using Amplex red with succinate as the substrate. SOD (40 U/ml) was added to convert all superoxide to hydrogen peroxide. Resorufin formation was measured at an excitation wavelength of 545 nm and a production wavelength of 590 nm. Hydrogen peroxide production was calculated with a standard curve and was normalized to the muscle fiber bundle dry weight.

### Plasma cytokine levels

To measure the plasma levels of TNFα, IL-1β and IL-6 blood samples were collected at experiment endpoint. ~200 μl of blood was collected and centrifuged at 5,000 rpm for 10 min at 4°C. The plasma levels of TNFα (RAB0477; Sigma-Aldrich, St. Louis, MO, USA), IL-1β (BMS6002; Thermo Fisher Scientific) and IL-6 (RAB0308; Sigma-Aldrich) were determined via ELISA using the manufacturer’s instructions.

### Western blot analysis

Heart and diaphragm muscle samples were homogenized 1:10 (wt/vol) in 5 mM Tris and 5 mM EDTA with a protease inhibitor cocktail (Sigma-Aldrich). Supernatant was collected and muscle protein content was assessed via Bradford method. Proteins were separated by electrophoresis, transferred to nitrocellulose membranes and incubated with a primary antibody directed against citrate synthase (Abcam, Cambridge, MA, USA), VDAC (Santa Cruz Biotechnology, Santa Cruz, CA, USA), αII-spectrin (Santa Cruz Biotechnology), LC3B (Cell Signaling Technologies, Danvers, MA). GAPDH (heart) and α-Tubulin (diaphragm) were used to normalize for protein loading and transfer. Images were acquired using Pierce ECL2 chemiluminescent substrate (Thermo Fisher Scientific) and the G: Box imaging system (Syngene, Frederick, MD, USA) and were analyzed using ImageJ software (NIH).

### RNA isolation, cDNA synthesis and RT-PCR

Total RNA was isolated from heart and diaphragm muscle samples using Trizol-based methods as previously described [52]. cDNA was synthesized from 1 μg RNA and oligo dT primers using the RETROscript First Strand Synthesis Kit (Thermo Fisher Scienctific) according to manufacturer’s instructions. cDNA was added to a PCR reaction for real time-PCR using Taqman chemistry and a 7300 real-time PCR system (Applied Biosystems). Relative quantitation of gene expression was performed using the relative standard curve method. 18S (reference gene), MuRF1 and BNIP3 mRNA transcripts were assayed using predesigned mouse primer and probe sequences commercially available (Thermo Fisher Scientific).

### Statistical analysis

A student’s *t*-test was used to compare tumor weight between groups. All other dependent measures were analyzed by two-way analysis of variance (ANOVA). When main effects were detected a Tukey HSD (honestly significantly different) test was performed *post hoc* to determine differences between the means. Significance was established at *p* < 0.05 and all values are reported as mean ± standard error.

## CONCLUSIONS

Cardiac and skeletal muscle mitochondrial dysfunction has been reported in cancer patients and preclinical models of cancer cachexia and is consistent with functional changes involving muscle contractile properties and cardiac function. While a growing body of research has begun to elucidate the biological processes associated with tumor-induced muscle pathologies, including mitochondrial dysfunction, to date no therapeutic interventions exist to combat the deleterious effects that cancer has on the functional capacity of the cardiorespiratory system, and thus the elevated mortality resulting from heart failure and ventilatory insufficiency. In the current study, we provide evidence that administration of the cell-permeable synthetic mitochondrial peptide SS-31 has therapeutic efficacy in combating C26 cachexia. This represents a novel approach to treating cancer cachexia, and warrants future work to determine effective timing and dosing strategies.

## SUPPLEMENTARY MATERIALS


