# Supplementary material for: Pharmacological targeting of mitochondrial function and reactive oxygen species production prevents colon 26 cancer-induced cardiorespiratory muscle weakness
Source: Oncotarget. 2020 Sep 22;11(38):3502–14. doi: 10.18632/oncotarget.27748 (PMC7517961; doi:10.18632/oncotarget.27748)
Supplement: Supplementary file 1 [file oncotarget-11-3502-s001.pdf]

Pharmacological targeting of mitochondrial function and reactive oxygen species production prevents colon 26 cancer-induced cardiorespiratory muscle weakness

SUPPLEMENTARY MATERIALS

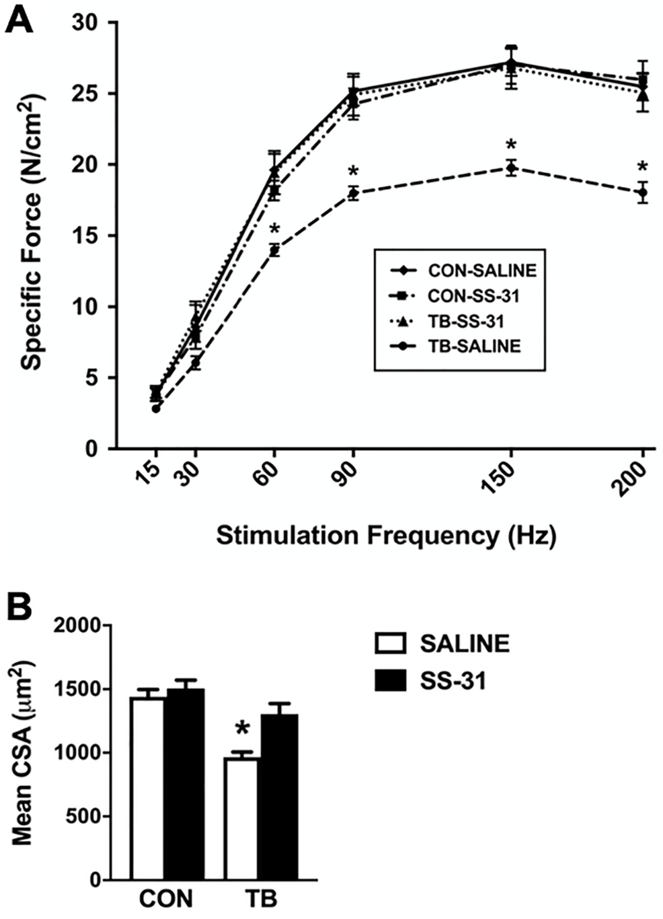

Supplementary Figure 1: Extensor digitorum longus (EDL) muscle function and fiber size. EDL muscle (**A**) force-frequency response and (**B**) cross-sectional area for control (CON) and tumor-bearing (TB) mice treated with saline (SALINE) or SS-31. Values are presented as means ± SEM. *significantly different versus all groups ($p < 0.05$).
